# WGCCRR: a web-based tool for genome-wide screening of convergent indels and substitutions of amino acids

**DOI:** 10.1093/bioadv/vbae070

**Published:** 2024-05-24

**Authors:** Zheng Dong, Chen Wang, Qingming Qu

**Affiliations:** State Key Laboratory of Cellular Stress Biology, School of Life Sciences, Xiamen University, Xià-Mén, Fú-Jiàn 361102, China; State Key Laboratory of Cellular Stress Biology, School of Life Sciences, Xiamen University, Xià-Mén, Fú-Jiàn 361102, China; State Key Laboratory of Cellular Stress Biology, School of Life Sciences, Xiamen University, Xià-Mén, Fú-Jiàn 361102, China

## Abstract

**Summary:**

Genome-wide analyses of proteincoding gene sequences are being employed to examine the genetic basis of adaptive evolution in many organismal groups. Previous studies have revealed that convergent/parallel adaptive evolution may be caused by convergent/parallel amino acid changes. Similarly, detailed analysis of lineage-specific amino acid changes has shown correlations with certain lineage-specific traits. However, experimental validation remains the ultimate measure of causality. With the increasing availability of genomic data, a streamlined tool for such analyses would facilitate and expedite the screening of genetic loci that hold potential for adaptive evolution, while alleviating the bioinformatic burden for experimental biologists. In this study, we present a user-friendly web-based tool called WGCCRR (Whole Genome Comparative Coding Region Read) designed to screen both convergent/parallel and lineage-specific amino acid changes on a genome-wide scale. Our tool allows users to replicate previous analyses with just a few clicks, and the exported results are straightforward to interpret. In addition, we have also included amino acid indels that are usually neglected in previous work. Our website provides an efficient platform for screening candidate loci for downstream experimental tests.

**Availability and Implementation:**

The tool is available at: https://fishevo.xmu.edu.cn/.

## 1 Introduction

Organismal genomes have been sequenced at an unprecedented speed (Rhie *et al.* 2021, Lewin *et al.* 2022), and these new data have fueled research efforts to unravel the connections between genomic diversity and phenotypic variation. Consequently, comparative genomic approaches such as “Forward Genomics” (Hiller *et al.* 2012, Roscito *et al.* 2022) and convergent evolution screening (Parker *et al.* 2013, Foote *et al.* 2015, Hu *et al.* 2017, Kowalczyk *et al.* 2019, Marcovitz *et al.* 2019, Xu *et al.* 2021, Kowalczyk *et al.* 2022) have emerged to identify genetic loci that potentially contribute to the observed adaptive traits. Although it is still under debate to what extent convergent morphological evolution is caused by convergent molecular evolution (Stern 2013, Storz 2016), experimental validations have confirmed that some convergent amino acid substitutions have probably contributed to certain adaptive changes, for example the echolocation in mammals (Liu *et al.* 2014, 2018, He *et al.* 2021), the fin size in flying fish (Daane *et al.* 2021) and hypoxia adaptation in mammals native to the Tibetan Plateau (Xu *et al.* 2021). Thus, the study of adaptive convergent evolution offers a unique opportunity to explore the genotype–phenotype relationships (Hao *et al.* 2019, Hu *et al.* 2023). However, it is important to consider that factors such as phylogenetic history, population demography, genetic (developmental) constraints, and epistasis all contribute to the likelihood of molecular parallelism (Kryazhimskiy *et al.* 2014, Rosenblum *et al.* 2014).

Nevertheless, most genomic screening studies remain as hypotheses pending rigorous experimental tests, which are considered the gold standard for establishing causality (Storz 2016, Zhang 2023). Despite some successful cases mentioned above, other adaptive convergent traits have been found to be caused by different loci, although in the same gene (Natarajan *et al.* 2015, 2016). In addition, experimental validation of identified loci can serve as a starting point for understanding the physiological functions of relevant proteins. If these loci are found to be associated with human diseases, experiments could potentially offer mechanistic explanations (Yamaguchi *et al.* 2023). Indeed, uncovering the functional mysteries embedded in organismal genomes necessitates greater involvement of experimental biologists. Existing tools for convergent molecular screening are either not designed for genome-wide analysis (e.g. Yuan *et al.* 2019, Barteri *et al.* 2023) or require extensive bioinformatic manipulations (e.g. Duchemin *et al.* 2023, Fukushima and Pollock 2023, Morel *et al.* 2024). Here, we introduce WGCCRR (Whole Genome Comparative Coding Region Read), a user-friendly web-based tool, designed to facilitate researchers in screening potential functional loci of evolutionary importance for convergent evolution or lineage-specific loci (see Section 2). Our website aims to alleviate the burden of accessing genomic data and streamline the screening process for evolutionary developmental biologists who may have limited access to programming and computing resources. This will accelerate experimental tests on genetic loci that are potentially relevant to adaptive or morphological evolution. The website can also serve as a preliminary screening tool for studying molecular convergent/parallel evolution in coding regions, providing a reference before conducting more specific analyses.

## 2 Methods

**Figure 1.**
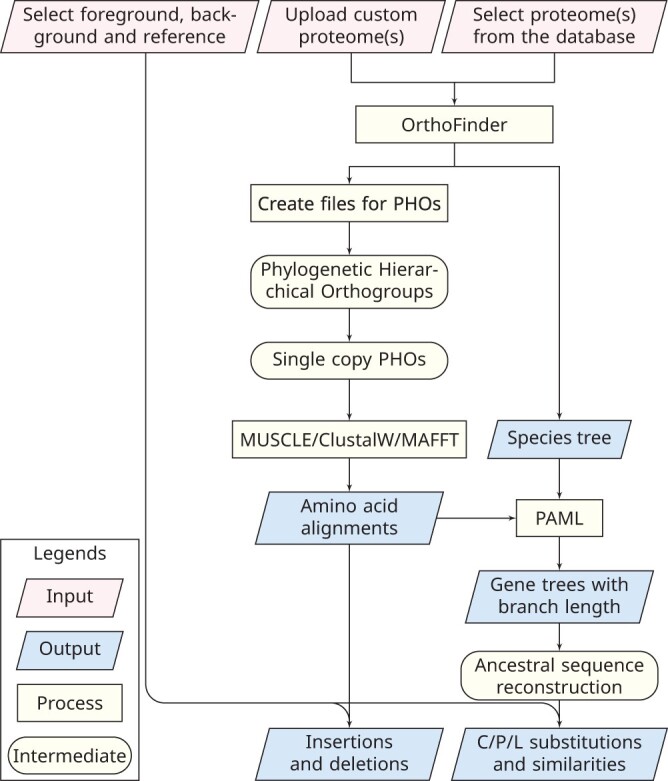
Workflow of the WGCCRR. The analysis starts from proteome files of the species being investigated, and the exported results include convergent (C)/parallel (P) and lineage-specific (L) amino acid changes in designated foreground species.

**Figure 2.**
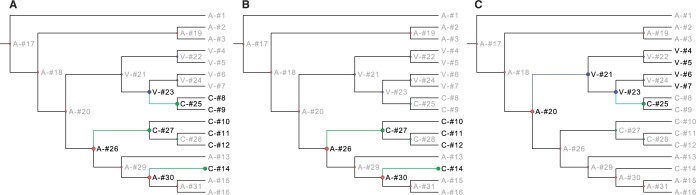
Different types of substitutions identified by the WGCCRR. (A) Changes from A (the ancestral state) to C (the derived state) from node #26 to node #27 and node #30 to node #14, and another change from V to C in node #23 to node #25. The convergent substitutions are denoted by green lines. (B) Changes from A to C occurred from node #26 to node #27 and node #30 to node #14. The parallel substitutions are denoted by green lines. (C) An ancestral change from A to V occurred from node #20 to node #21. This change is shared by all foreground species except two background species #8 and #9, which have experienced another change from V to C from node #23 to node #25. The Lineage-specific amino acid substitutions are denoted by blue lines. Foreground species and concerned ancestral species are represented in thicker nodes.

The WGCCRR web tool focuses solely on protein-coding genes, utilizing proteomes based on the longest transcripts of the species under investigation as input files (Fig. 1). Amino acid insertions, deletions, and substitutions that are shared by all and only foreground species (which exhibit similarities in certain phenotypic traits of interest) are identified. In this study, we adhere to the previously well-defined definitions of convergent and parallel evolution of amino acids (Zhang and Kumar 1997, Storz 2016). Convergent substitutions at a site refer to independent changes from different ancestral amino acids to the same derived amino acid. When more than two foreground branches are selected, we allow different foreground branches to share the same ancestral amino acid, if no single ancestral amino acid is shared by all foreground branches (Fig. 2A). Parallel substitutions at a site refer to independent changes from the same ancestral amino acid to the same derived amino acid (Fig. 2B). Specifically, when such substitutions are inherited from the common ancestor of all (but not limited to) foreground species, they are defined as lineage-specific substitutions. Background species are also allowed to share such an ancestral substitution, as long as each of them has diverged and differs from foreground species at this site (Fig. 2C). While the contribution of amino acid indels to adaptive evolution is less understood compared to substitutions (Savino *et al.* 2022), we have incorporated the analysis of amino acid indels into the tool. It has been demonstrated that amino acid indels can also play a role in adaptive evolution, as seen in the recurrent evolution of resistance to cardiotonic steroids (Mohammadi *et al.* 2022). The identified loci are categorized as either convergent/parallel or lineage-specific sites, depending on the definition of the foreground species. We will use the echolocation evolution in mammals and the evolution of seahorses as two cases to illustrate how to use the website in these two different scenarios.

## 3 Results

The WGCCRR tool is primarily developed based on OrthoFinder (Emms and Kelly 2017, 2018, 2019) and PAML (Yang 2007) algorithms. It identifies foreground-specific amino acid mutations in foreground species that differ from those in background species. Foreground species are defined as those exhibiting similarities in certain phenotypic traits of interest, either derived from common ancestry or due to convergent evolution. Conversely, background species are defined as those exhibiting different phenotypes.

To initiate an analysis, users need to upload canonical proteome files of the species under investigation. For convenience, we have prepared proteome files that include the longest transcripts of all vertebrates in the Ensembl database (as of release 109) (Martin *et al.* 2023) using a custom Perl script. Once the proteome files are uploaded or selected, users are required to assign each species as either foreground or background. Additionally, one reference species from the foreground group should be designated. It is recommended to choose a model species with a well-assembled and well-annotated genome, as this facilitates result interpretation and identification of genes and proteins with foreground-specific amino acid changes.

The workflow of WGCCRR (Fig. 1) begins by utilizing OrthoFinder to identify single-copy orthologs and construct the phylogenetic tree of the input species. The single-copy orthologs extracted from the Phylogenetic Hierarchical Orthogroups (PHOs) directory are further processed and exported. Each single-copy ortholog sequence file is named after the gene symbol of the reference species. Furthermore, the website also provides an export of the Species Tree, which represents the evolutionary relationships among the input species.

Once the single-copy orthologs are extracted, WGCCRR provides users with a choice among three widely used multiple sequence alignment (MSA) programs: ClustalW (Larkin *et al.* 2007), MUSCLE (Edgar 2022), and MAFFT (Katoh *et al.* 2002, Katoh and Standley 2013). After the alignment process, a custom Perl script within WGCCRR analyzes the MSA files to identify shared amino acid insertions, deletions, and substitutions in the selected foreground species. In the MSA files, sites with retained gaps are examined for deletions and insertions. A shared amino acid deletion is defined as a site where all background species possess the same amino acid, while all foreground species have a gap at the aligned site. Conversely, a shared amino acid insertion is defined as a site where all background species have a gap, while all foreground species share the same amino acid at the aligned site (Supplementary Fig. 1).

**Figure 3.**
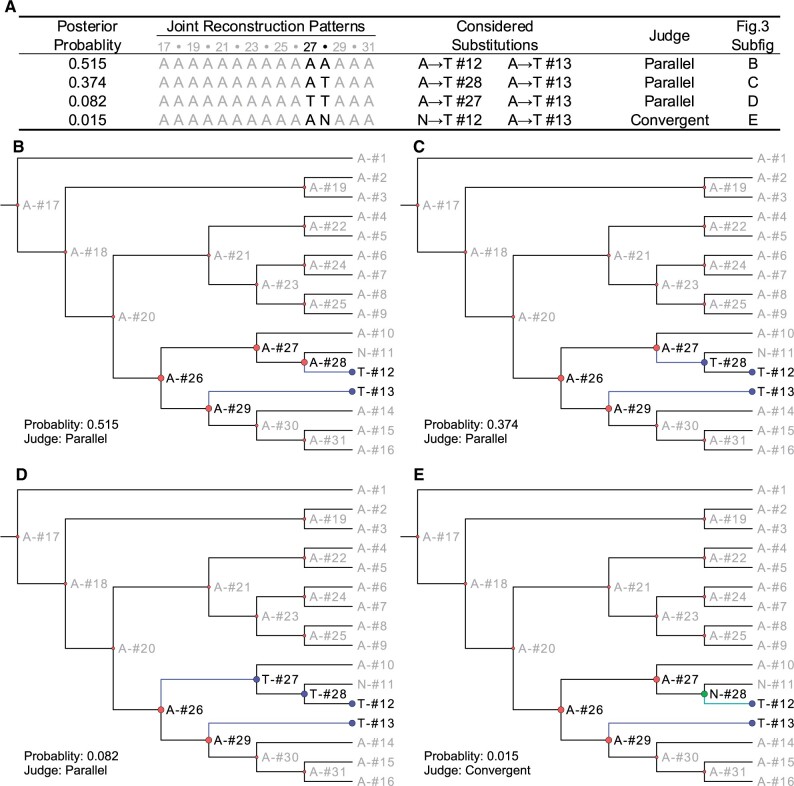
A case of a SINGLE-SITE substitution used to explain the significance of considering the sub-optimal states. (A) Four joint reconstruction patterns by PAML and their posterior probabilities. Note that the rows of the reconstruction pattern indicate different states of the joint reconstruction, while the columns indicate different ancestral nodes as numbered from 17 to 31 in such states. Each of these states is visualized separately in (B–E). All ancestral nodes, except nodes #27 and #28, are reconstructed to be A with high posterior probabilities, strongly supporting a substitution from A at node #29 to T at the tip #13. (B) The “optimal” status, which supposes that node #28 is A and parallel substitution occurs between node #28 to node #12 and node #29 to node #13, is of high uncertainty (only with 0.515 posterior probability). (C) The “suboptimal” status supposes an A at node #27 and a T at node #28, supporting parallel substitution that occurs between node #27 to node #28 and node #29 to node #13 and a divergent substitution from node #28 to tip #11. (D) Another condition supposes both nodes #27 and #28 to be T, supporting parallel substitution that occurs between node #26 to node #27 and node #29 to node #13. All three scenarios (B–D) suggest that tips #12 and #13 share a parallel substitution, although when node #12 experienced the substitution remains unresolved. (E) The probability is low for node #28 to be N, in which case node #12 and node #13 experienced a convergent substitution.

To identify shared amino acid substitutions, WGCCRR utilizes the codeml program from PAML (Yang 2007). This involves removing all gaps in the alignments and reconstructing ancestral sequences at each node of the phylogenetic tree to infer convergent/parallel amino acid substitutions in the foreground species (Fig. 2A and B). This strategy has been employed in previous studies (e.g. Thomas and Hahn 2015, Zou and Zhang 2015, Liu *et al.* 2018). Furthermore, WGCCRR identifies lineage-specific amino acid substitutions when all foreground species inherit the amino acid state from their common ancestor (Fig. 2C). It is worth noting that sub-optimal states of ancestral amino acids with slightly lower posterior probabilities are also considered (Fig. 3). This is necessary to avoid false negative results (Fig. 3), as relying solely on optimal states may lead to incomplete findings (Yang 2007).

The final exported files from WGCCRR include the following:

- Species tree from OrthoFinder.
- Aligned sequence files for all single-copy orthologs (under Single_Copy_PHO_Sequences/directory).
- Four tables:
  - count.tsv lists the number of shared amino acid substitutions, insertions, and deletions for each gene.
  - scan-ins.tsv provides the position of each foreground-specific insertion.
  - scan-del.tsv provides the position of each foreground-specific deletion.
  - scan-sub.tsv provides detailed information about each foreground-specific substitution beside its position. The first column displays the amino acids at a particular site among all species, following the same order as in the fasta files under Single_Copy_PHO_Sequences/. The posterior probabilities, which indicate the likelihood of a certain site undergoing convergent, parallel, or lineage-specific amino acid changes, are also provided. Additionally, pairwise similarities of the 21 amino acid residues centered on each shared substitution site are calculated. If the average pairwise similarity is greater than 0.7, while the lowest pairwise similarity is greater than 0.35, the corresponding sites are labeled as “well aligned fragments.” Otherwise, they are labeled as “poorly aligned fragments” (He *et al.* 2021). It is recommended that users consider sites with posterior probabilities greater than 0.95 and labeled as “well aligned fragments” for further downstream experiments.

## Case study 1: convergent evolution of echolocation in bats and toothed whales

The independent acquisition of echolocation in different lineages of mammals has been well documented (Thomas *et al.* 2004). Previous studies have identified convergent amino acid substitutions in the *Prestin* gene in both bats and toothed whales (Li *et al.* 2010, Liu *et al.* 2010), as well as similar convergent signals in other auditory genes (Shen *et al.* 2012). The first genome-wide screening of molecular convergence in echolocation identified over 100 loci that may have contributed to its convergent evolution, including several sites in the *Prestin* gene (Parker *et al.* 2013). However, subsequent scrutiny of that study suggested that there is no genome-wide convergence signal underlying the convergent evolution of echolocation (Thomas and Hahn 2015, Zou and Zhang 2015). Nevertheless, experimental studies have demonstrated that many of the identified convergent amino acid substitutions are functionally relevant to echolocation, such as the *Prestin* gene (Liu *et al.* 2014, 2018), or indirectly relevant to echolocation behavior, like the fast-twitch muscle fiber genes (Lee *et al.* 2018). This highlights the importance of experimental validation of screening results to determine the extent to which convergent evolution of echolocation is driven by convergent molecular evolution, a topic crucial for understanding the predictability of evolution (Stern 2013).

**Figure 4.**
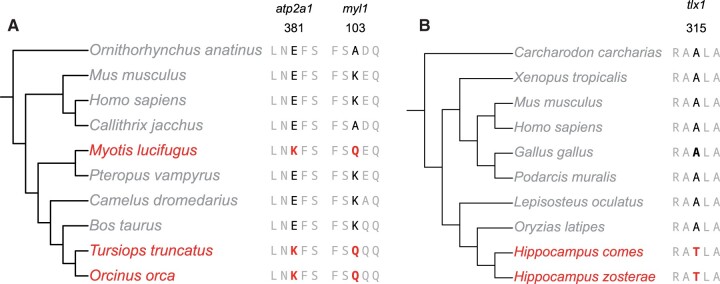
The phylogenetic trees and multiple sequence alignments of the two case studies. (A) Screening of convergent/parallel amino acid changes related to echolocation in bats (*Myotis lucifugus*) and toothed whales (*Tursiops truncates* and *Orcinus orca*) (B) Screening lineage specific changes of amino acids in *Hippocampus*. The species highlighted in red are the foreground species.

For our analysis, we utilized WGCCRR to examine a dataset similar to the one used by Thomas and Hahn (2015). We selected *Myotis lucifugus* as a bat species and *Orcinus orca* and *Tursiops truncatus* as toothed whale species as foreground species. Additionally, seven other mammals including *Pteropus vampyrus*, *Ornithorhynchus anatinus*, *Bos taurus*, *Camelus dromedarius*, *Callithrix jacchus*, *Homo sapiens*, and *Mus musculus* were designated as background species (Fig. 4A). The proteome files, except for *O. orca*, were retrieved from the Ensembl database, and all the necessary input files can be found on our WGCCRR website. We could identify all the convergent sites reported in the *Prestin* gene (Liu *et al.* 2018) and the fast-twitch muscle fiber genes (Lee *et al.* 2018), except for *Myh2*, which was not included in the list of single-copy orthologs. Improvements in single-copy ortholog identification will be valuable contributions to our web-based tool in the future.

## Case study 2: *Hippocampus*-specific amino acid changes

Syngnathidae include four groups of fish that exhibit significant phenotypic diversification: pipefishes, pygmy pipehorses, seahorses, and seadragons (Stiller *et al.* 2022). The monophyly of syngnathids has been supported in recent phylogenomic studies (Hamilton *et al.* 2017, Longo *et al.* 2017, Santaquiteria *et al.* 2021, Stiller *et al.* 2022). Sequencing of syngnathid genomes has prompted the investigation of the genetic basis underlying the phenotypic evolution, such as the male brooding pouch (Lin *et al.* 2016, Small *et al.* 2016, Zhang *et al.* 2020), leaf-like appendages (Qu *et al.* 2021, Small *et al.* 2022), and loss of teeth and spleen (Liu *et al.* 2022).

We used WGCCRR to analyze the *Hippocampus*-specific amino acids using *Hippocampus comes* and *Hippocampus zosterae* as the foreground species. Ten other species, including *H.sapiens*, *M.musculus*, *Gallus gallus*, *Podarcis muralis*, *Xenopus tropicalis*, *Carcharodon carcharias*, *Lepisosteus oculatus*, and *Oryzias latipes* were designated as background species (Fig. 4B). Using *H.zosterae* as the reference species, 5140 single-copy orthologs have been identified. *Hippocampus*-specific insertions, deletions, and substitutions have been identified in 2728, 584, and 5085 genes, respectively. It is noteworthy that amino acid indels occur less frequently compared to substitutions, indicating that indels are less likely to occur during evolution than substitutions (Chen *et al.* 2009). As expected, a *Hippocampus*-specific amino acid substitution in *tlx1* has been identified (see case results in the website), which probably contributes to the loss of spleen in seahorses (Liu *et al.* 2022).

## 4 Discussion

As demonstrated in the previous case studies, screening for such foreground-specific amino acid changes can yield a large number of gene candidates for experimental validation, which may not always be feasible. Furthermore, it is important to note that the identified loci may not necessarily involve adaptive evolution but could be the result of neutral evolution (Thomas and Hahn 2015, Zou and Zhang 2015). Although some studies have shown that there may not be a genome-wide convergent signal compared to neutral evolution, functional enrichment tests have confirmed that certain echolocation-related genes have indeed undergone adaptive convergent evolution (Marcovitz *et al.* 2019). These enrichment tests can serve as useful references for selecting loci for experimental validation from the large pool of candidates. Furthermore, users are advised to approach with caution the single-copy orthologs extracted from the PHOs directory according to the results obtained from OrthoFinder, as their accuracy may not be guaranteed.

The exported results from our tool also provide information about the conservation of the region surrounding the identified convergent substitution loci, which can be used as a reference prior to conducting experiments. Predictive tools such as SIFT (Kumar *et al.* 2009) and PROVEAN (Choi and Chan 2015) can be utilized to assess the potential functional effects of the amino acid substitutions. However, it is essential to consider different genomic backgrounds and the influence of epistasis (Jordan *et al.* 2015), as the ultimate validation lies in well-designed experiments. For example, the loss of the spleen in seahorses is attributed to a single amino acid substitution in the *tlx1* gene, despite the fact that this gene is neither positively selected nor evolving at a faster rate in seahorses (Qu *et al.* 2021). In other cases, examining whether genes with convergent substitutions are under positive selection can help users evaluate candidate loci before conducting experiments (Daane *et al.* 2015, 2021). It is important to note that the relevance of certain amino acid changes to adaptive phenotypic changes is independent of the evolutionary rates of the genes that have undergone these changes. Furthermore, predictive models such as SIFT (Kumar *et al.* 2009) may indicate that the same missense mutation in humans is not disease related. Improvements in these predictive models may help address such challenges, but experiments will continue to be the gold standard for testing hypotheses (Zhang 2023).

## Supplementary Material

vbae070_Supplementary_Data

## Data Availability

The tool is available at https://fishevo.xmu.edu.cn/. The data used in the case studies can be retrieved from the website.
