# Supplementary figures and images for: WGCCRR: a web-based tool for genome-wide screening of convergent indels and substitutions of amino acids
Source: Bioinform Adv. 2024 May 24;4(1):vbae070. doi: 10.1093/bioadv/vbae070 (PMC11132816; doi:10.1093/bioadv/vbae070)

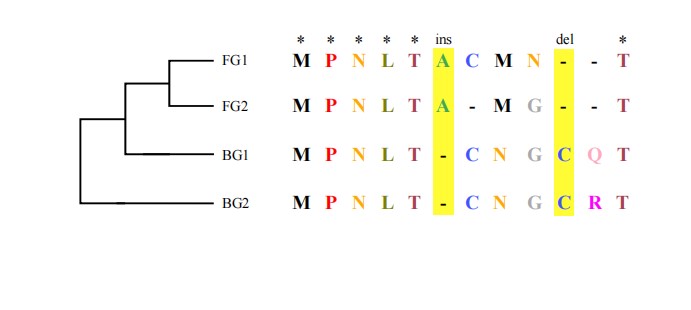

Supplement: vbae070_Supplementary_Data [file vbae070_supplementary_data.jpeg]
